# First Structural View of a Peptide Interacting with the Nucleotide Binding Domain of Heat Shock Protein 90

**DOI:** 10.1038/srep17015

**Published:** 2015-11-24

**Authors:** Swetha Raman, Meetali Singh, Utpal Tatu, Kaza Suguna

**Affiliations:** 1 Molecular Biophysics Unit, Indian Institute of Science, Bangalore, India; 2 Department of Biochemistry, Indian Institute of Science, Bangalore, India

## Abstract

The involvement of Hsp90 in progression of diseases like cancer, neurological disorders and several pathogen related conditions is well established. Hsp90, therefore, has emerged as an attractive drug target for many of these diseases. Several small molecule inhibitors of Hsp90, such as geldanamycin derivatives, that display antitumor activity, have been developed and are under clinical trials. However, none of these tested inhibitors or drugs are peptide-based compounds. Here we report the first crystal structure of a peptide bound at the ATP binding site of the N-terminal domain of Hsp90. The peptide makes several specific interactions with the binding site residues, which are comparable to those made by the nucleotide and geldanamycin. A modified peptide was designed based on these interactions. Inhibition of ATPase activity of Hsp90 was observed in the presence of the modified peptide. This study provides an alternative approach and a lead peptide molecule for the rational design of effective inhibitors of Hsp90 function.

Hsp90 is a ubiquitous molecular chaperone, which plays a crucial role in maturation and activation of a wide array of client proteins under normal and stress-related conditions. Many of its well established clientele such as signalling kinases, transcription factors, steroid hormone receptors etc., play central roles in an array of cellular processes like cell signalling, cell survival, proliferation and apoptosis. Many of these proteins are oncogenic and necessary for tumorigenesis^1^. Many of the client proteins have also been relevant in the onset of neurodegenerative disorders and viral infections^2^. Blocking the function of Hsp90 causes proteosomal degradation of the oncogenic client proteins, leading to the control of the growth of cancer cells. Several Hsp90 inhibitors have been shown to exhibit antitumor, anti-parasitic and antiviral activity, thereby making Hsp90 an effective drug target for cancer and many other infections^3^,^4^,^5^,^6^,^7^,^8^.

Hsp90 consists of three distinct functional domains: the N-terminal ATP binding domain (NTD), the middle domain and the C-terminal dimerization domain. The first crystal structure of the NTD was determined for human Hsp90 in complex with the antitumor drug geldanamycin^9^. Based on this structure, it was initially predicted that geldanamycin competitively inhibits binding of unfolded proteins to this domain. However, the true function of NTD came into light when the structure of NTD from yeast Hsp90 in complex with ATP/ADP was determined^10^, and limited sequence homology between Hsp90 and other ATP dependent enzymes like type II topoisomerases and the MutL mismatch repair proteins was recognized^11^. This led to the identification of key residues involved in ATP binding, implicating the action of geldanamycin as a competitive inhibitor of ATP binding. Due to the characteristic fold of the NTD, Hsp90 is classified under GHKL family of ATPases^12^, which includes DNA gyrase, histidine kinase and MutL, among others. Inhibition of ATP binding and hydrolysis by natural product inhibitors like geldanamycin and radicicol have demonstrated the dependence of Hsp90 function on ATPase activity^13^,^14^. This makes the NTD a potential target domain for designing various Hsp90 inhibitors and drug molecules which currently include geldanamycin and radicicol derivatives, and purine based inhibitors. In spite of the development of drugs such as 17-AAG, NVP-AUY922, IPI504 and STA-9090, which are in clinical trials^15^,^16^, it is still a challenge to find more effective Hsp90 inhibitors that are more soluble and less toxic, as well as to explore new avenues to approach this problem. Hsp90 of *Dictyostelium discoideum* (HspD) with 64% sequence identity with the human enzyme serves as a good model system to study the structural and functional aspects of Hsp90. We had earlier reported the activity studies of the full length protein and the crystal structure of the N-terminal domain of HspD (HspD-NTD) in the native form, at a resolution of 2.7 Å^17^ (PDB code: 4XKK). We have also determined the crystal structure of HspD-NTD in the presence of ATP, AMPPCP, AMPPNP, ATP-γS and geldanamycin. While attempting to obtain the structure of the native NTD at a higher resolution, we observed a heptapeptide fragment occupying the ATP binding pocket of this domain and making specific interactions with the binding site residues. We modified the sequence of this peptide and designed a new hexapeptide for better interaction with HspD-NTD. ATPase activity of Hsp90 was evaluated in the presence of this modified peptide. This is the first reported structure of a peptide bound to the NTD of Hsp90 and here we present this structure, which provides a basis for the development of peptide-based inhibitors of Hsp90.

## Results

Crystal structures of HspD-NTD in the native form and in complex with different ligands have been determined. As all the structures were refined using data that extend to 1.8 Å resolution or better, a reliable comparison of ligand-protein interactions could be carried out. We also observe a peptide bound to the nucleotide binding pocket of HspD-NTD. The structure of this complex will be discussed in detail and compared to the structures of NTD in complex with other ligands.

### Structure of NTD in complex with peptide

**Figure 1:**
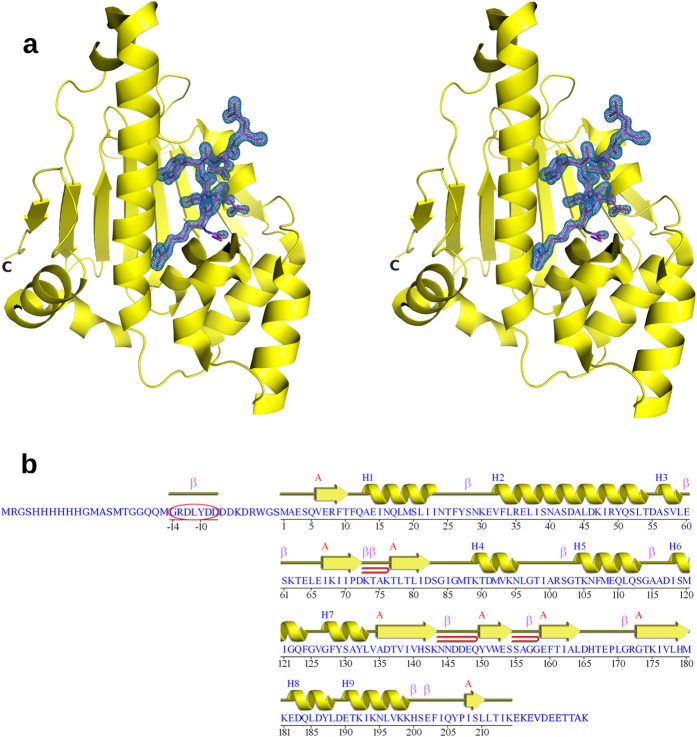
Structure of HspD-NTD in complex with a heptapeptide, GRDLYDD. (**a**) Stereo image of the peptide bound at the nucleotide binding site of HspD-NTD. The 2F_o_-F_c_ map for the peptide was contoured at 1.5 σ. (**b**) Sequence of HspD-NTD construct used for expression. Secondary structural elements as observed in the crystal structure are marked. The heptapeptide sequence bound at the ATP binding site is circled.

The structure of HspD-NTD is similar to the other reported structures of equivalent domain from homologous Hsp90s consisting of a typical Bergerat fold^11^ of two layer α/β sandwich motif and a central cavity forming the nucleotide binding site with the hydrophobic face of the β-sheet as the base and the helices as walls. During the initial stages of model building and refinement of the native HspD-NTD structure, a long continuous density was observed in the ATP binding pocket. A tyrosine residue could be clearly identified from the density. It was possible to build residues on either side of the tyrosine and extend it to a short peptide (Fig. 1a). This was an unexpected result as no peptide was added to the protein. Careful examination of the density and the sequence of the HspD-NTD clone revealed a heptapeptide, Gly-Arg-Asp-Leu-Tyr-Asp-Asp, which was present in the pRSET A vector sequence between the N-terminal hexahistidine tag and the NTD protein sequence, used for the expression (Fig. 1b). The structure refined well on addition of the peptide. The first 214 of a total of 225 residues of HspD-NTD and the bound peptide were clearly visible in the electron density. The peptide appeared to be cleaved and released from the fusion protein as the distance between the N-terminus of the protein and the C-terminus of the peptide is 40 Å and no electron density appeared on either side of the peptide.

From the structure of NTD of human Hsp90 reported in complex with geldanamycin, it was initially speculated that geldanamycin inhibits Hsp90 by competing with the binding of the substrate protein to NTD due to general similarities between the geldanamycin ansa ring and a peptide^9^. It was hypothesized that a five amino acid polypeptide in a bent conformation can trace the geldanamycin ansa ring backbone with its side chains corresponding to the different groups of the ansa ring. A peptide model was also proposed, consisting of a Tryptophan residue whose position corresponds to the carbamate group of geldanamycin and can interact with the Asp93 residue of the NTD of human Hsp90. There are reports of development of peptidomimetic inhibitors like Shepherdin and its derivatives against Hsp90^18^, where Shepherdin was predicted to dock into ATP binding site. In another study, sansalvamide, a cyclic depsipeptide, and its derivatives were shown to inhibit Hsp90 activity^19^,^20^. However, this natural product was shown to bind to a different location on Hsp90^19^. The molecular basis for a peptide interacting with the NTD of Hsp90 is not known as no crystal structure with a bound peptide was available until now.

### Peptide-NTD interactions

**Figure 2:**
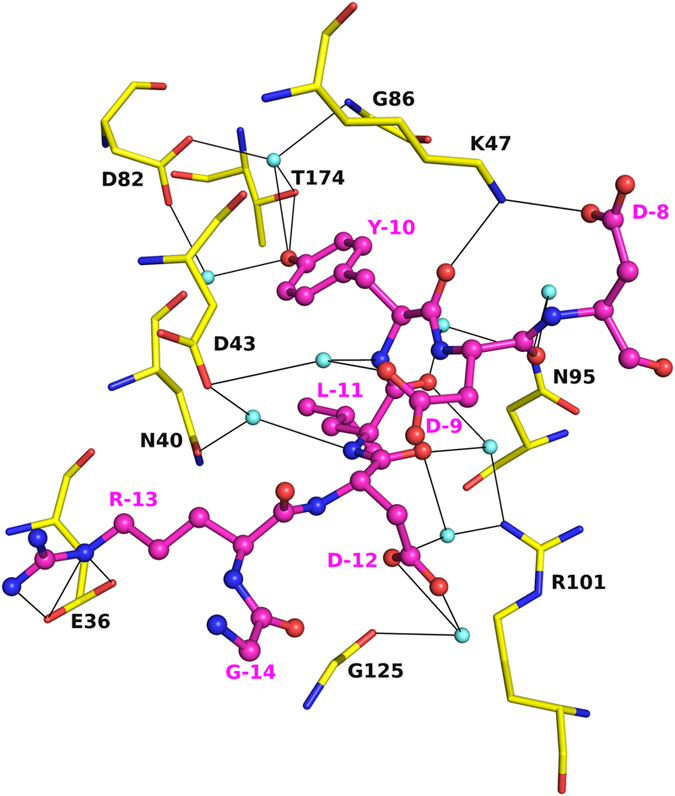
Peptide-protein interactions. Interactions of peptide GRDLYDD (pink) with residues of HspD-NTD lining the pocket (yellow) and bound solvent molecules (cyan spheres). Hydrogen bonds are shown as black lines. The peptide makes hydrogen bonds (with T147, K47), several water-mediated interactions and forms two salt-bridges (R-13∙∙∙E36 and D-8∙∙∙K47) with the protein.

The peptide Gly-Arg-Asp-Leu-Tyr-Asp-Asp (numbered from −14 to −8 starting from Gly) occupying the positions of ATP/geldanamycin was found to make direct and many solvent mediated interactions with the protein (Fig. 2). The Tyr-10 residue is buried in the pocket and interacts with the residues involved in interacting with the base of the nucleotide. The side chain hydroxyl group of Tyr-10 of the peptide makes a direct hydrogen bond with the side chain of Thr174 and water mediated interactions with the side chains of Asp82, Thr174 and the backbone N atom of Gly86. The backbone O atom of Tyr-10 forms a hydrogen bond with the side chain of Lys47 and the N atom makes a water mediated interaction with the side chain of Asp43. The side chain of Asp-12 makes water mediated interactions with the O atom of Gly125 and the side chain of Arg101. The N atom of Leu-11 makes water mediated hydrogen bonds with the side chains of Asn40 and Asp43. The O atom of Leu-11 makes water mediated hydrogen bonds with the side chains of Asn95 and Arg101. The side chain of Asp-9 interacts with the side chain of Asp43 through a water molecule, while its O atom makes a water mediated hydrogen bond with the side chain of Asn95. The peptide makes two salt bridge interactions with the protein between Asp-8∙∙∙Lys47 and Arg-13∙∙∙Glu36. These two salt bridges impart additional strength to the interactions between the peptide and the protein.

**Figure 3:**
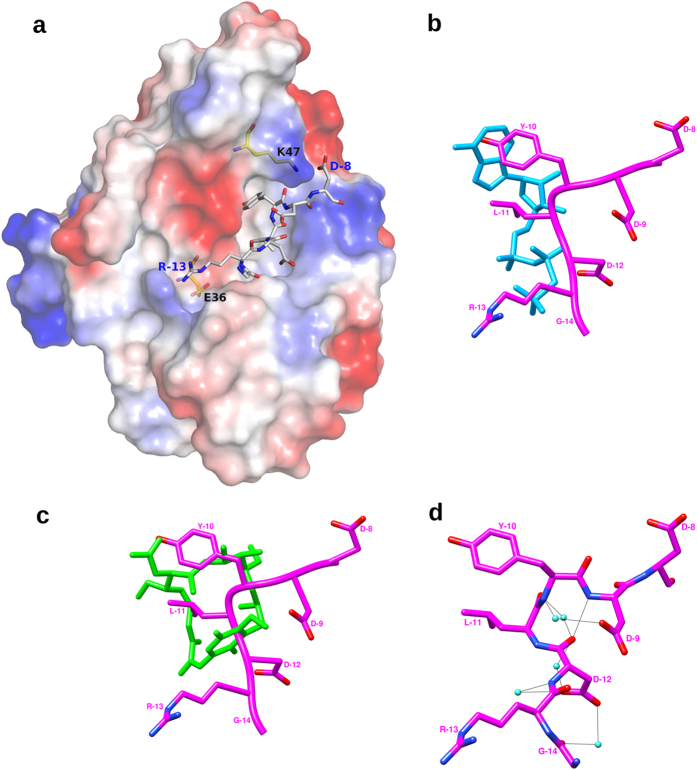
Interacting nature and conformation of the peptide. (**a**) Electrostatic surface representation of HspD-NTD with bound peptide, indicating the nature of the complementary interaction of the anchoring residues R-13 and D-8, of the peptide with the protein. Residues of HspD-NTD, E36 and K47, that are involved in these interactions are also shown. Overlay of peptide (pink) on (**b**) AMPPCP (blue) and (**c**) Geldanamycin (green). (**d**) Internal interactions stabilizing the peptide conformation.

The peptide has a basic amino acid (Arginine) on one side, an acidic one (Aspartate) on the other and hydrophobic residues in the middle. The hydrophobic Tyrosine and Leucine residues of the peptide are buried in the pocket, while the flanking charged residues interact with residues of complementary charge at the edges of the pocket (Fig. 3a). The peptide occupies similar position as the nucleotide or geldanamycin in the pocket (Fig. 3b,c) and is observed to be more extended, and forms more solvent mediated interactions.

In addition to making various contacts with the protein, the peptide is also internally stabilized by forming hydrogen bonds between its residues. The peptide has an internal Type I β-turn with the formation of Asp-12∙∙∙Asp-9 hydrogen bond. The O atom of Asp-12 also forms a water mediated hydrogen bond with the O atom of Leu-11 (Fig. 3d).

### Comparison of the structures of HspD-NTD in complex with peptide, nucleotide and geldanamycin

**Figure 4:**
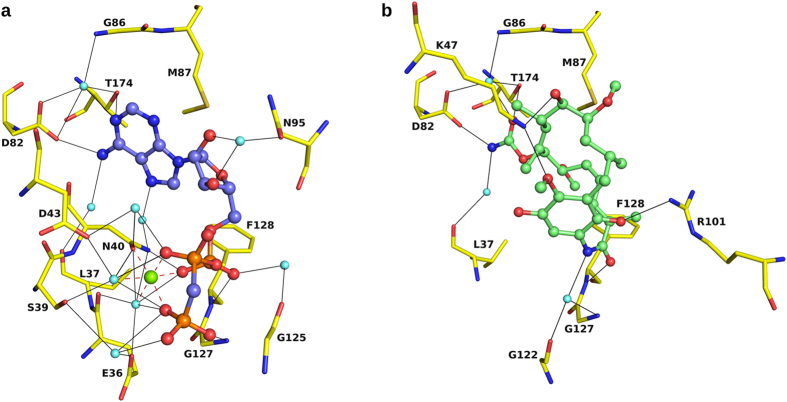
Interaction of different ligands with HspD-NTD. (**a**) Interaction of AMPPCP (blue) with the protein (yellow). The green sphere represents Mg^2+^ and water molecules are shown as cyan spheres. Hydrogen bonds are shown as black lines and metal coordination as red dashed lines. (**b**) Interaction of geldanamycin (green) with the protein.

We compared the peptide-bound structure of HspD-NTD with the structures of other complexes that we determined. While the γ-phosphate in ATP, AMPPNP and ATPγS were disordered, it appeared only in AMPPCP. We compared the interactions made by the peptide with those made by AMPPCP and geldanamycin (Fig. 4 and listed in Supplementary Table S1). We observed that water molecules replace the phosphate groups in case of the peptide complex, which allows the peptide to extend its interaction with the protein through waters. Asp82, which is an essential residue for recognition and binding of the nucleotide^13^,^21^, interacts with all the three ligands. It forms direct as well as solvent mediated interactions with the base of the nucleotide and carbamate group of geldanamycin. In case of the peptide, it forms a water-mediated interaction with the side chain of Tyr-10 residue. Replacing this residue with a tryptophan is likely to make the interactions stronger as the indole group could mimic the adenine base and can interact directly with Asp82. The residues of the ATP lid segment (Gly97-Phe128), with its glycine rich loop that positions γ-phosphate for hydrolysis, also makes several interactions with all the three ligands. Lys47 makes two direct hydrogen bonds with geldanamycin, but does not show any interaction with AMPPCP. However, it forms a salt-bridge and a hydrogen bond with the peptide. On the other side of the peptide, Arg-13 makes a salt bridge with Glu36 residue, which is implicated in catalysis^13^ and makes solvent mediated interactions with the phosphate groups of the nucleotide, but shows no interaction with geldanamycin. The two residues Arg-13 and Asp-8 of the peptide, through salt bridges, anchor the peptide to the surface of Hsp90.

### Inhibition of ATPase activity of HspD by a peptide

The crystal structure of HspD-NTD shows binding of the peptide Gly-Arg-Asp-Leu-Tyr-Asp-Asp to the ATP binding pocket. The contacts made by the peptide and Hsp90 are similar to those made by Hsp90 with ATP or its competitive inhibitor geldanamycin with additional stabilizing salt bridges. Binding of the peptide to the NTD should block the binding of ATP to the pocket, thereby causing a decrease in the ATPase activity. Therefore, ATPase activity of HspD was checked in the presence of 100 μM-13.5 mM concentration range of the peptide. However, no significant change in the ATPase activity was observed in the presence of the peptide. Based on the interactions observed in the crystal structure, the peptide was modified with a view to enhance the interactions of the peptide with the protein. The N-terminal Glycine was deleted, as it did not form any specific interactions with the protein. The Tyrosine residue was replaced by a Tryptophan, in order to mimic the interactions made by the base of the nucleotide. Asp-12, which forms a water mediated interaction with Arg101, was replaced by a Glutamate residue with the possibility of it forming a direct hydrogen bond with the protein. The effect of the resulting hexapeptide Arg-Glu-Leu-Trp-Asp-Asp, on the ATPase activity of HspD was examined.

**Figure 5:**
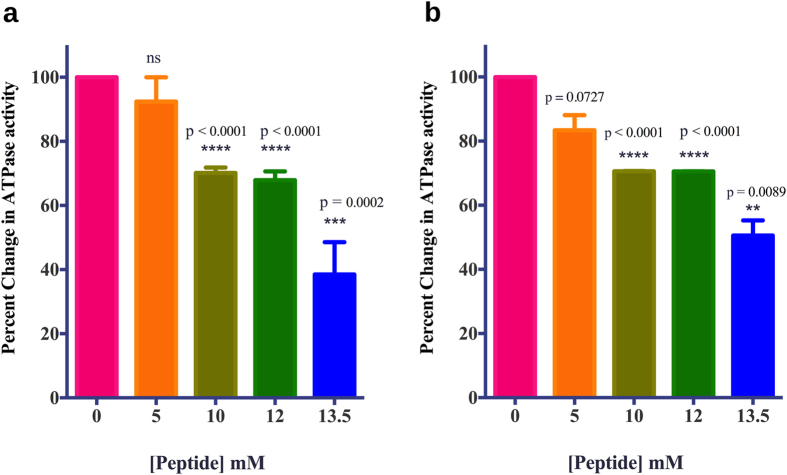
Inhibition of HspD ATPase activity with peptide RELWDD. (**a**) Percent inhibition of ATPase activity of HspD (2 μM) with increasing molar concentration of the peptide RELWDD (n = 5). At the highest concentration of 13.5 mM of the peptide, only 38.50 ± 10.11% activity is observed. Data set was evaluated by ANOVA with p value < 0.0001. (**b**) ATPase inhibition of new construct of HspD by peptide RELWDD (n = 2). At highest concentration of 13.5 mM of the peptide, 50.60 ± 4.68% ATPase activity is observed. Data set was evaluated by ANOVA with p value = 0.002. Bars indicate the mean ± SEM. Individual series were evaluated using two-tailed t-test.

ATPase assay for HspD was carried out in presence of varying concentrations of the peptide and fixed ATP concentration of 500 μM. In presence of the peptide, there is a significant dose dependent inhibition of ATPase activity of HspD, starting from 5 mM concentration of the peptide (Fig. 5a). At a concentration of 10 mM peptide, 30% inhibition of ATPase activity was observed, and upon further increase of the concentration to 13.5 mM, 60% inhibition in activity was observed, thus suggesting that, in principle, this peptide can bind to Hsp90 at its ATP binding pocket and inhibit the ATPase activity of Hsp90.

The modified peptide showed weak ATPase inhibition, whereas the peptide bound to HspD-NTD in the crystal structure showed no inhibition whatsoever. To check whether the low inhibition is the consequence of the peptide already present in the binding pocket, as it is part of the fusion protein, we cloned the full length protein in pET-28a vector which does not have this peptide sequence in the construct. However, the inhibition results did not change (Fig. 5b) indicating that either the observed inhibitory values are intrinsic to the peptides or the binding pocket is empty in both the constructs in solution. Bio-Layer Interferometry experiments were carried out to check the binding of the peptide RELWDD. Weak binding of the peptide to HspD-NTD, which was also cloned in pET-28a vector, was observed with a K_D_ in the lower micromolar range (see Supplementary Fig. S1). In addition, progressive decrease in the binding of the protein to the peptide was observed in the presence of increasing concentrations of ATP (see Supplementary Fig. S2), indicating that ATP competes with the peptide to bind to HspD-NTD. The peptide can be further modified to improve the binding efficacy, thereby reducing the inhibitory concentration required to inhibit Hsp90 function.

### Implications on human Hsp90

**Figure 6:**
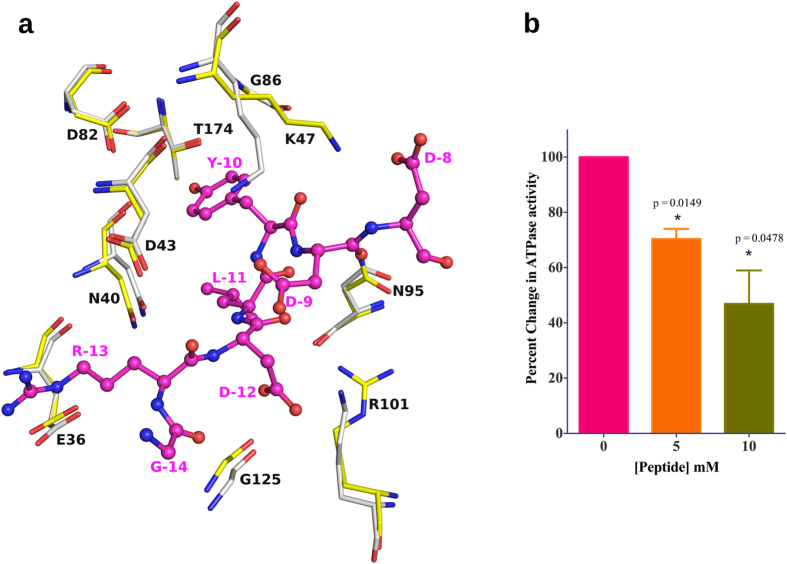
Interaction of peptide with human Hsp90. (**a**) Comparison of residues interacting with the peptide (pink) in HspD-NTD (yellow) and NTD of human Hsp90 (grey). (**b**) Inhibition of ATPase activity of human Hsp90 in the presence of peptide RELWDD. The plot shows inhibition of human Hsp90 (2 μM) by increasing molar concentration of peptide RELWDD (n = 2). At 10 mM peptide concentration only 46.91 ± 12.05% activity is remaining. Data set was evaluated by ANOVA with p value = 0.032. Bars indicate the mean ± SEM. Individual series were evaluated using two-tailed t-test.

A comparison of HspD-NTD with that of the human homologue (PDB code: 3T10)^22^ shows that the residues interacting with the peptide are conserved in the two proteins, except that Arg101 in HspD, is replaced by Lys112 in the human Hsp90 (Fig. 6a). Lys47 of HspD-NTD is observed to be in a different rotamer conformation as compared to Lys58 of the human isoform, due to the former’s interaction with the residues of the peptide. Lys58 of the human Hsp90 is likely to adopt a similar conformation in the presence of a similar peptide. The water molecules interacting with Asn40 and Asp82 in the case of HspD-NTD are also found to be conserved in human Hsp90-NTD structure. Hence, it can be suggested that the peptide is also capable of binding to the NTD of human Hsp90 in a manner observed in the HspD-NTD structure. In order to validate this, ATPase assay was performed for human Hsp90 in the presence of the modified peptide. The hydrolysis of ATP was observed to decline in the presence of increasing concentrations of the modified peptide. At 5 mM concentration of the peptide, Arg-Glu-Leu-Trp-Asp-Asp, 30% inhibition of ATPase activity was observed, and the activity was inhibited up to 55% at 10 mM peptide concentration (Fig. 6b). The human homologue was found to be more prone to inhibition by the peptide, as it showed a larger decrease in its activity, at a lower concentration of the peptide, as compared to HspD. This observation that the peptide binds to the NTD and inhibits its ATPase activity paves way for the development of new class of peptide-based inhibitors for Hsp90.

## Discussion

In this study, the structure of the NTD of Hsp90 from *D. discoideum* was analyzed in the native form and in complex with several ligands. We crystallized HspD-NTD in complex with ADP, AMPPCP, AMPPNP, ATPγS and geldanamycin. The protein-ligand interactions were found to be similar to the ones reported in the structures of NTD from various organisms. We have observed for the first time a heptapeptide bound at the nucleotide binding site of NTD, occupying the binding pocket as the other ligands and making specific interactions with the protein. A thorough analysis of the protein-peptide interactions and the solvent molecules in the binding pocket was carried out and compared with the interactions of other ligands with the NTD. Most of the residues involved in interaction with the peptide and the other ligands were common and they interact either directly or through water molecules. In addition, the two residues at the N- and the C-terminal ends of the peptide make strong electrostatic interactions with residues on the surface of the protein. The peptide comes from the vector used to express the protein. As the peptide failed to inhibit the ATPase activity of HspD, we designed a new hexapeptide, based on the interactions observed in the structure, by modifying the sequence of the bound peptide. A tyrosine was changed to tryptophan to mimic the natural substrate ATP. This and another change from an Asp residue to a Glu residue were incorporated anticipating that two water mediated interactions of the residues, Tyr and Asp with the protein will become direct interactions thus increasing the binding strength of the peptide. This modified peptide RELWDD was found to inhibit the ATPase activity of HspD and the human Hsp90, confirming its binding at the ATP binding site. More potent inhibitors could be designed and tested with this peptide as the lead compound.

Natural products such as geldanamycin and radicicol have played a key role in unravelling the biological function of Hsp90 and in identifying it as a molecular target for cancer drugs^23^,^24^. These compounds act as competitive inhibitors, docking into the ATP-binding site in the NTD of Hsp90^25^. These natural product inhibitors have been proved to be too toxic and unsuitable for clinical trials due to high reactivity and low stability. Several different derivatives of geldanamycin and radicicol have been developed, which show better solubility and less toxicity^15^,^26^,^27^. Other inhibitors such as BIIB021 and PU-H71 are based on the purine scaffold of the natural nucleotide ligands^28^,^29^,^30^. Peptidomimetic compounds like Shepherdin exhibit anti-leukaemic activity and are predicted to interact with NTD^18^. The crystal structure of the peptide bound to the NTD of Hsp90 and the inhibition of ATPase activity of Hsp90 from *D. discoideum* and its human homologue, by the modified peptide presented here, provide a structural platform for the development of peptide-based inhibitors to this protein. There is a potential to design peptides with better binding affinities based on the NTD-peptide interactions observed in the structure presented here.

## Methods

### Cloning and protein purification

The genes for HspD and HspD-NTD were cloned into pRSET A vector, using the following primers: 5′ CTCCGCCTGGGATCCAAAATGGCTGAATCACAAGTTG 3′ as forward primer and 5′ CTCATTCTCGAGCTAGTCGACTTTTTCCATT 3′ as reverse primer for HspD as described previously^17^, and 5′ CTCCGCCTGGGATCCAAAATGGCTGAATCACAAGTTG 3′ as forward primer and 5′CTCATTCTCGAGCTATTTGGCAGTGGTTTC 3′ as reverse primer for HspD-NTD, containing BamHI and XhoI sites (underlined) in forward and reverse primers, respectively. The proteins, HspD and HspD-NTD, were expressed as hexa histidine tagged fusion proteins in *E. coli* Rosetta strain. The cells were grown in Terrific broth and induced with 1 mM isopropyl β-D-1-thiogalactopyranoside at 15 °C for 15 hours. The proteins were purified by nickel-NTA affinity chromatography. The column was washed with imidazole gradient ranging from 5 mM to 30 mM in 50 mM Tris, pH 7.5, 500 mM NaCl, buffer. The proteins were finally eluted with 300 mM imidazole, followed by gel filtration with Sephacryl S-200 HR column and stored in 25 mM Tris and 200 mM NaCl, pH 7.5 buffer. The clone for human Hsp90 in pRSET A vector was expressed in *E. coli* Rosetta strain, and the protein was purified to homogeneity by nickel-NTA affinity chromatography, as described previously^31^ and dialysed to exchange buffer containing 40 mM Tris-Cl, pH 7.5, 100 mM KCl and 5 mM MgCl_2_ for the ATPase assay. For the new construct the gene for HspD and HspD-NTD were cloned in pET-28a vector, which lacks the bound peptide sequence. The proteins were expressed and purified using the same protocol as described above.

### Peptides

The two peptides, GRDLYDD and RELWDD purchased from Custom peptide synthesis, USV Limited, Mumbai, were synthesized by Fmoc chemistry on solid support and purified using RP-HPLC.

### Crystallization and data collection

The crystallization of the NTD construct was carried out using the hanging drop method with 2 μl of the protein and 2 μl of the precipitant solution. The concentration of protein used for crystallization trials was 15 mg/mL. Crystals appeared in two weeks in a condition similar to the previously crystallized native form of HspD-NTD^17^, consisting of 0.1 M HEPES pH 7.0–8.0 and 10-30% PEG3350. The crystals diffracted to a resolution of 1.2 Å at BM14 beamline of ESRF.

Two data sets were collected: (i) a high resolution data with an exposure time of 4 seconds, 0.5° oscillation and a crystal-to-detector distance of 102 mm, and (ii) a low resolution data with an exposure time of 1 second, 1° oscillation and a crystal-to-detector distance of 190 mm. The data sets were scaled using SCALA of CCP4 suite^32^.

### Structure solution and refinement

The crystal belonged to space group P2_1_. The structure of the complex was determined by molecular replacement using PHASER^33^ with HspD-NTD (PDB code: 4XCJ) as search model (Z score of 32 and LLG of 1449). During the initial stages of model building, a long extra density was observed in the binding pocket. Automated model building in ARP/wARP^34^ and *Phenix.autobuild*^35^ fitted a short Alanine polypeptide in the extra density. Subsequent model building and solvent additions were performed by manual inspection using Coot^36^ and the structure was refined using the program REFMAC5^37^. The structure refined well upon the addition of the peptide with side chains to an R_work_ 21.0% and R_free_ of 23.0%. After the addition of solvent molecules and several cycles of refinement, the refinement converged to an R_work_ and R_free_ of 18.5% and 21.0%, respectively. The data collection and refinement details are given in Table 1. The figures were made using the program PyMol (DeLano Scientific LLC.) and UCSF Chimera^38^.

### ATPase activity

In order to check the effect of the peptide binding on the ATPase activity of HspD and human Hsp90, purified proteins (2 μM) were incubated with varying peptide concentrations in 40 mM Tris–Cl buffer, pH 7.4, containing 100 mM KCl and 5 mM MgCl_2_, at room temperature for half hour. ATPase reaction was carried out in the presence of fixed ATP concentration of 500 μM at 37 °C for one hour. γ-^32^P-ATP with a specific activity of 0.55 Ci/mmol was used as a tracer. ATP hydrolysis was monitored on a PEI- cellulose TLC plate. Fractional cleavage of ATP was calculated for each peptide concentration. ATPase activity of Hsp90 alone was considered 100%, and percent change in ATPase activity was plotted against peptide concentration.

### Binding studies using Bio-Layer Interferometry

The binding of the peptide RELWDD to HspD-NTD construct lacking the linker peptide was measured by Bio-Layer Interferometry using an Octet RED96 instrument (ForteBio, Inc.). The peptide was immobilized onto Amine Reactive Second-Generation (AR2G) Biosensors. Binding studies were performed by dipping the biosensors in varying concentrations of HspD-NTD. An equal number of unliganded sensors was used as control, to account for non-specific binding. The traces were processed using ForteBio Data Analysis Software, ver. 8.0.3.5, exported and fit globally using Biaevaluation 3.0.1. A simple 1:1 Langmuir interaction model was used for fitting the data.

For the competitive binding assay, ATP at two concentrations of 30 μM and 300 μM was added to HspD-NTD with a fixed concentration of 300 μM and the binding of the protein to the peptide was monitored.

## Additional Information

**Accession codes:** The structures of HspD-NTD complexes have been submitted to the Protein Data Bank with the following PDB codes: 4XE2 for peptide complex, 4XCJ for ADP complex, 4XC0 for AMPPCP complex, 4XCL for ATPγS complex, 4XD8 for AMPPNP complex and 4XDM for geldanamycin complex.

**How to cite this article**: Raman, S. *et al.* First Structural View of a Peptide Interacting with the Nucleotide Binding Domain of Heat Shock Protein 90. *Sci. Rep.* **5**, 17015; doi: 10.1038/srep17015 (2015).

## Supplementary Material

Supplementary Information

## Figures and Tables

**Table 1:** Data collection and refinement statistics.

Data Collection	
Space Group	P2_1_
Cell Parameters	
a (Å)	41.91
b (Å)	46.63
c (Å)	59.76
β (˚)	107.12
Wavelength (Å)	0.8865
Resolution Range (Å)	57.14–1.20
	(1.26–1.20)
R_merge_ (%)	9.9 (40.4)
Average I/σ(I)	9.6 (3.1)
Completeness (%)	96.3 (94.3)
Multiplicity	5.6 (4.1)
**Refinement Statistics**	
No. of reflections used	
Total	66263
Working set	62904
Test set	3359
R_work_ (%)	18.5
R_free_ (%)	21.0
No. of atoms	
Protein atoms	1836
Solvent atoms	279
Average B-factor (Å^2^)	17.0
RMS deviation from ideal values	
Bond length (Å)	0.008
Bond angle (˚)	1.22
Residues in Ramachandran plot (%)	
Most favoured	98
Additionally allowed region	2
Values in parentheses correspond to the last resolution shell	
