# Supplementary material for: First Structural View of a Peptide Interacting with the Nucleotide Binding Domain of Heat Shock Protein 90
Source: Sci Rep. 2015 Nov 24;5:17015. doi: 10.1038/srep17015 (PMC4657054; doi:10.1038/srep17015)
Supplement: Supplementary Information [file srep17015-s1.pdf]

Supplementary Information

First Structural View of a Peptide Interacting with the Nucleotide Binding Domain of Heat Shock Protein 90

Swetha Raman[1,+], **Meetali Singh**[2,+], **Utpal Tatu**[2,*] and **Kaza Suguna**[1,*]

[1]Molecular Biophysics Unit, and [2]Department of Biochemistry, Indian Institute of Science, Bangalore, India

[+]These authors contributed equally to this work

*Corresponding authors

tatu@biochem.iisc.ernet.in and suguna@mbu.iisc.ernet.in

Table S1. Comparison of the interacting residues of HspD-NTD with different ligands (direct interactions are shown in bold).

Residue	AMPPCP	Geldanamycin	Peptide
Glu36<br>OE1<br><br>OE2<br><br>O	 α- and γ-PO_4<br><br><br><br>α- and γ-PO_4		 **NE of Arg-13**<br><br>**NE and NH2 of Arg-13**
Leu37<br>O	 N6	 N2	
Ser39<br>OG	 β- and γ-PO_4		
Asn40<br>OD1<br><br>ND2	 α-, β-, and γ-PO_4<br>**Mg^{2+}**<br>**α-PO_4**<br>N7		 N of Leu-11
Asp43<br>OD2	 β- and γ-PO4		 OD1 Asp-9<br>N of Tyr-10<br>N of Leu-11
Lys47<br>NZ		 **O5 and O7**	 **OD1 of Asp-8**<br>**O of Tyr-10**
Asp82<br>OD2<br>OD1	 **N6** and N1<br>N1	 **N2**<br>O4	 OH of Tyr-10<br>OH of Tyr-10
Gly86<br>N	 N1	 O4	 OH of Tyr-10

Asn95<br>OD1<br>ND2	O2' and O3'		O of Leu-11<br>O of Asp-9
Arg101<br>NH2		**O9**	O of Leu-11<br>OD1 and O of Asp-12
Gly122<br>O		N1	
Gly125<br>O	α-PO_4		OD1 and OD2 of Asp-12
Gly127<br>N	**γ-PO_4**	N1	
Phe128<br>N	**α-PO_4**	**O1**	
Thr174<br>OG1	**N1**<br>N1	**O4**<br>O4	**OH of Tyr-10**<br>OH of Tyr-10

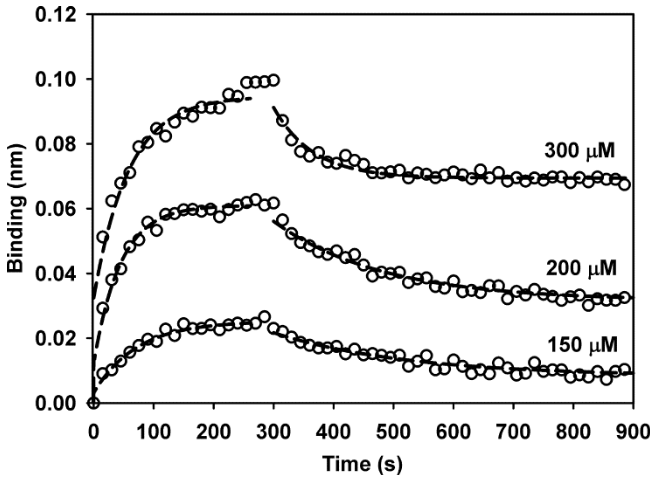

Figure S1. Binding of HspD-NTD to the peptide RELWDD using Bio-layer Interferometry. The plot shows the overlay of the binding kinetics of HspD-NTD to the peptide at different concentrations of the protein. The kinetic parameters (mean ± standard deviation) for the binding are k_{on} = 90.57 ± 46.60 $M^{-1}s^{-1}$, k_{off} = 2.1 ± 0.4 × 10^{-3} s^{-1} and K_D = 28.2 ± 14.6 µM.

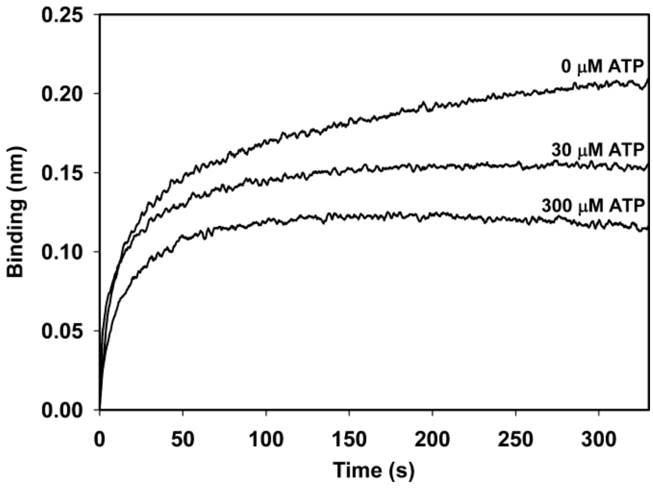

Figure S2. Binding of HspD-NTD to the peptide RELWDD in the presence of ATP using Bio-layer Interferometry.

The plot shows the overlay of the binding profiles of HspD-NTD to the peptide in the presence of different concentrations of ATP. A decrease in the association signal of the protein to the peptide is observed with increasing ATP concentrations.
